# Supplementary material for: A retrospective observational study of enhanced recovery after surgery in older patients undergoing elective colorectal surgery
Source: PLoS One. 2020 May 8;15(5):e0232857. doi: 10.1371/journal.pone.0232857 (PMC7209103; doi:10.1371/journal.pone.0232857)
Supplement: S1 Table — (DOCX) [file pone.0232857.s001.docx]

**S1 Table.** Standard ERP in the University Hospitals Leuven for colorectal surgical patients in 2017

| **ERP component** | | **ERP in the University Hospitals Leuven (2017)** |
| --- | --- | --- |
| 1 | Preadmission counselling | Pre-hospital visit to ERP nurse, anesthesiologist and surgeon.  For patients with planned stoma, stoma-education is provided pre-operatively. |
| 2 | Preoperative optimization | No routine protocol for preoperative optimization/prehabilitation. |
| 3 | Preoperative bowel preparation | Patients undergoing colonic operations do not receive mechanical bowel preparation (they may receive a rectal enema). In patients undergoing rectal surgery mechanical bowel preparation is performed (if no obstruction). |
| 4 | Preoperative fasting  and preoperative carbohydrate drink | Last meal and drinks are served the evening before the operation. Patients are allowed (light) solid foods until 6 hours and clear fluids until 3 hours before the operation. A carbohydrate drink is provided 3 hours prior to the operation. |
| 5 | Pre-anesthetic medication | Sedative or anxiolytic premedication is not given routinely. |
| 6 | Thromboprophylaxis | All patients receive prophylaxis with low molecular weight heparin.  Compression stockings are not used routinely. Pneumatic compression is not used. Patients are stimulated to move (exercises in bed in an instruction leaflet, early mobilization out of bed). Patients with increased risk for venous thromboembolism are given prolonged thromboprophylaxis (3 weeks). |
| 7 | Antimicrobial prophylaxis | All patients receive prophylaxis with antibiotics per protocol. |
| 8 | Standard anesthesia protocol | Balanced anesthesia (with an inhaled anesthetic) or a total intravenous anesthesia is used (according to the anesthetists preference) with short or intermediate acting analgesics, anesthetics and muscle relaxants. The anesthetist monitors electrocardiogram, blood pressure (non-invasive or invasive depending on the extent of the intervention and the comorbidity of the patient), oxygen saturation, capnometry and capnography, temperature, neuromuscular monitoring, ventilation parameters, and aims for a zero fluid balance. Goal-directed fluid therapy is not routinely used (sometimes in open surgery). |
| 9 | PONV prophylaxis | PONV risk is screened preoperatively with the Apfel score and PONV prophylaxis is administered per protocol (dexamethasone ± dehydrobenzperidol ± ondansetron) in patients with a risk ≥20%. If PONV is present, multimodal treatment is given per protocol (ondansetron, dexamethasone, dehydrobenzperidol, domperidone). |
| 10 | Laparoscopy and modifications of surgical access | Minimal invasive surgery is first choice. |
| 11 | Nasogastric tubes | Nasogastric tubes inserted during surgery are removed in the operating room. |
| 12 | Preventing intraoperative hypothermia | Intraoperative normothermia is maintained by using a bear-hugger and by warming intravenously administered fluids. |
| 13 | Perioperative fluid management | Postoperatively intravenous fluids are administered per protocol (preferably balanced crystalloids). Fluid overload, as well as dehydration, are avoided (and closely monitored). Goal directed fluid therapy is used in extensive open surgical procedures and patients with severe cardiac comorbidity. Other patients receive intravenous fluids at 2-3 ml/kg/hour and compensation for blood loss with crystalloids. Vasopression is considered in epidural-induced hypotension (if the patient is normovolemic). Intravenous lines are discontinued as soon as possible (usually by POD 3, provided that the PCEA or PCIA has been removed). |
| 14 | Drain peritoneal cavity | No routine drainage of the peritoneal cavity in colonic surgery. In rectal surgery drainage is allowed. |
| 15 | Urinary catheter | The urinary catheter is removed routinely by POD 1 in laparoscopic colonic surgery and by POD 3 in open colonic or in rectal surgery, or after cessation of the PCEA. |
|  |  |  |
| 16 | Ileus prevention | Ileus is prevented by giving preference to laparoscopic surgery, by considering PCEA for open procedures, and by avoiding fluid overload and by avoiding routine nasogastric decompression. Chewing gum and mobilization for patients developing ileus, laxatives in patients developing constipation. |
| 17 | Multimodal opioid sparing postoperative analgesia | Patients receive a tailored analgesia protocol, composed of weak or strong opioids in combination with paracetamol and/or NSAIDs because of their opioid sparing effect. Patient-controlled epidural analgesia (PCEA) is routine in patients undergoing open surgery (if no contra-indication, such as anticoagulant therapy, severe back problems, infection of the puncture site, sepsis, or refusal by the patient). Patients undergoing laparoscopic surgery receive either PCEA (especially patients with an extensive history of Crohn’s or colitis ulcerosa), patient-controlled intravenous analgesia (PCIA) or patient-controlled oral analgesia with Sufentanil (PCOA). PCE/IA is removed on POD 1 (laparoscopic surgery), POD 3 (open surgery) or POD 5 (APER). Special consideration to the most appropriate therapy is given in all patients aged over 75. |
| 18 | Early nutrition | The Nutritional Risk Score (NRS-2002) is scored at admission to the hospital. Patients at risk for malnutrition are systematically seen by a dietician. Clear liquids are allowed on POD 0. Four to eight hours after surgery rusks are allowed. On POD 1 the patient receives a light breakfast (toast) and a light solid meal is allowed if this is well tolerated. From POD 2, the patient receives low-fiber solid meals. From POD 1, the meals are supplemented with nutritional supplement drinks. |
| 19 | Postoperative glycemic control | Blood glucose levels are monitored in diabetes patients. Insulin in is not given on the morning of surgery. Postoperatively patients with elevated glucose levels receive an insulin drip (usually until eating by mouth). |
| 20 | Early mobilization | The day of surgery patients sit shortly on the edge of the bed with assistance of a nurse. The day after surgery the aim is to sit at least for two hours in a chair and by POD 2 patients should be walking in the room with the nurse. By POD 3 patients should walk to the bathroom and outside the room (with assistance if needed). |
| LEGEND: APER: Abdomino-Perineal Excision of Rectum; ERP: enhanced recovery program; NRS: Nutritional Risk Score; NSAIDs: nonsteroidal anti-inflammatory drugs; PCEA: patient controlled epidural analgesia; PCIA: patient controlled intravenous analgesia; PCOA: patient controlled oral analgesia; POD: postoperative day; PONV: postoperative nausea and vomiting | | |
