# Supplementary material for: A retrospective observational study of enhanced recovery after surgery in older patients undergoing elective colorectal surgery
Source: PLoS One. 2020 May 8;15(5):e0232857. doi: 10.1371/journal.pone.0232857 (PMC7209103; doi:10.1371/journal.pone.0232857)
Supplement: S2 Table — (DOCX) [file pone.0232857.s002.docx]

**S2 Table.** Postoperative complications in detail

| **Complications** | **CD grade 1:**  **Minor/no treatment** | **CD grade 2:**  **Medication** | **CD grade 3:**  **Intervention** | **CD grade 4:**  **ICU admission** |
| --- | --- | --- | --- | --- |
| **RESPIRATORY SYSTEM** | Bronchitis (n=3) | Pneumonia (n=2) |  | Pneumonia (n=1) |
|  | Angina (n=4) |  |  |  |
|  | Non-infectious and non-cardiac respiratory deterioration (n=5) |  |  |  |
| **CARDIO-**  **VASCULAR**  **SYSTEM** |  | AF (n=2) | Reconversion AF (n=1) | AF (n=2) |
|  | Cardiac ischemia (n=1) |  |  | MI with MOF (n=1) |
|  | Fluid retention, no diuretics (n=2) | Fluid retention treated with diuretics (n=3) |  |  |
|  |  | Catheter infection (n=2) |  |  |
| **NEURO-**  **LOGICAL**  **SYSTEM** | Suspicion TIA (n=1) |  |  | Epileptic insult (n=1) |
|  | Syncope (n=1) |  |  | Syncope (n=1) |
|  | Altered mental status or confusion (n=12) | Confusion treated with medication (n=5) |  |  |
|  | Paresthesia upper limb (n=2) |  |  |  |
| **GASTRO-**  **INTESTINAL**  **SYSTEM** | Constipation/slow transit treated with laxatives or digitation (n=10) | Ileus or gastroparesis treated with NGT ± TPN ± prokinetics (n=14) |  |  |
|  | Diarrhea treated with IV fluids ± electrolytes ± loperamide (n=3) |  |  |  |
|  | Hiccups ± medical treatment (n=3) |  |  |  |
|  |  | Melena treated with transfusion (n=1) | Esophageal varices bleeding (n=1) |  |
| **URINARY**  **SYSTEM** | LUTS, no treatment (n=4) | UTI (n=9) |  |  |
|  | POUR with catheterization only (n=2) | POUR treated with alpha blockers (n=4) |  |  |
|  | Deterioration renal function (n=5) |  |  |  |
| **HEMATO-LOGICAL** |  | Transfusion for toxic or infectious anemia (n=4) |  |  |
| **SKIN** | Herpes labialis (n=1) |  |  |  |
|  | Pressure ulcer (n=4) |  |  |  |
|  | Rash/contact dermatitis (n=2) |  |  |  |
| **SURGICAL** | Wound infection, local therapy (n=3) | Wound infection treated with antibiotics (n=3) | Reoperation for anastomotic leak (n=4) | Septic shock due to anastomotic leak (n=2) |
|  | Ischemia stoma (n=1) | Organ/organ space infection treated with antibiotics (n=5) | Reoperation for persistent organ/organ space infection (n=1) |  |
|  | High output stoma + loperamide or octreotide (n=2) |  | Reoperation for iatrogenic perforation small intestine (n=1) | Septic shock due to iatrogenic perforation small intestine (n=1) |
|  | Hematoma abdominal wall (n=1) | *Transfusion for bladder haemorrhage*^1^ *(n=1)* | *Cystoscopic removal of blood clots*^1^ *(n=1)* |  |
|  |  |  | Percutaneous drainage seroma (n=1) |  |
| LEGEND: AF: atrial fibrillation; CD: Clavien-Dindo severity grade; IV: intravenous; LUTS: lower urinary tract symptoms; MI: myocardial infarction; MOF: multiple organ failure; n: number; NGT: nasogastric tube; POUR: postoperative urinary retention; TIA: transient ischemic attack; TPN: total parenteral nutrition; UTI: urinary tract infection; ^1^*due to combined urologic surgery* | | | | |
